# Important gender differences in psychosomatic and school-related complaints in relation to adolescent weight status

**DOI:** 10.1038/s41598-021-93761-0

**Published:** 2021-07-08

**Authors:** Samantha J. Brooks, Inna Feldman, Helgi B. Schiöth, Olga E. Titova

**Affiliations:** 1 Department of Neuroscience, Uppsala University, Uppsala, Sweden; 2 Faculty of Health, School of Psychology, Liverpool John Moores University, Liverpool, SE3 3AF UK; 3 Neuroscience Research Laboratory (NeuRL), Department of Psychology, School of Human and Community Development, University of the Witwatersrand, Johannesburg, South Africa; 4 Uppsala County Council, Uppsala, Sweden; 5 Department of Public Health and Caring Sciences, Uppsala University, Uppsala, Sweden; 6 Institute for Translational Medicine and Biotechnology, Sechenov First Moscow State Medical University, Moscow, Russia; 7 Unit of Medical Epidemiology, Department of Surgical Sciences, Uppsala University, Epihubben, Dag Hammarskjölds väg 14 B, 75185 Uppsala, Sweden

**Keywords:** Risk factors, Public health, Epidemiology, Human behaviour

## Abstract

Underweight or overweight in adolescence is linked to several adverse health outcomes. Less evidence exists about the association between weight status and school-related psychosocial characteristics in high income countries. We sought to investigate the relationship between weight status and psychosomatic and school-related complaints with a focus on gender differences. The study is a cohort of 18,462 adolescents (12–19 years; 51% girls) conducted in Sweden. The associations between weight status and psychosomatic and school-related complaints were estimated by binary logistic regression adjusted for several potential confounders. After correction for multiple testing, being underweight or overweight/obese was adversely associated with several psychosomatic and school-related complaints with significant differences between boys and girls. Specifically, underweight boys had higher odds to have psychosomatic complaints than normal-weight boys, while no such associations were observed among underweight girls. Overweight/obese (vs. normal-weight) boys had higher odds to complain about headache, pain in the back/hips, and feeling low. Overweight/obese (vs. normal-weight) girls were more likely to complain about feeling low, anxious/worried and having difficulty in falling asleep (P ≤ 0.01). In relation to school-related complaints (e.g., being bullied at school and academic failure), greater associations were observed for overweight/obese girls and boys than for underweight adolescents compared with normal-weight peers.

## Introduction

Overweight and obesity among children and adolescents has received attention in recent decades as a major global public health problem, associated with adverse physical and mental health outcomes^1–3^. Concomitantly, modern fashion standards and the ‘thin ideal’ may lead to body dissatisfaction among young people^4^, associated with weight loss and mental disorders including anxiety, depression and eating disorders such as anorexia nervosa^5^. Research in adolescents indicates that being underweight, overweight/obese or having body image distortion is associated with increased prevalence of depression, anxiety and suicidal behavior^6–10^. This association seems to differ across genders. For instance, in a cross-sectional study of 17-year-old adolescents a U-shaped association between body mass index (BMI) and depression scores was demonstrated in boys, with higher levels of depression among both underweight and overweight boys. However, a more complex association has been observed in girls; in support of the “fat and jolly hypothesis”, obese girls were less depressed than overweight, whereas underweight girls were more depressed than those of normal-weight^8^. Moreover, a prospective study of over two thousand U.S. adolescent boys revealed that average weight boys that perceived themselves as being either very underweight or overweight had higher prevalence of mental disorders such as depression, anxiety and suicidal behavior, compared to boys who viewed their weight as average^7^. Another study of middle school students demonstrated that females who perceived themselves as overweight were more likely to report suicidal thoughts and actions compared to normal-weight adolescents. Importantly, among male students in this study, perceptions of both being overweight and underweight were related to suicidal thoughts and actions^9^.

The relationship between being underweight or overweight and psychosomatic complaints (PSC) in adolescents is less studied. PSC refer to psychological and physical symptoms experienced by an individual with or without a defined diagnosis, such as anxiety, depression, headache and stomach ache. PSC have been considered as possible indicators of poor health among children and adolescents^11^. For instance, PSC have been previously associated with stress, poorer well-being and mental ill-health symptoms^12–14^. A Swedish study based on the Health Behaviour in School-aged Children survey (HBSC) showed increasing rates of mental health complaints among older adolescents, especially in girls^15^. It has been demonstrated that overweight and obese children and adolescents as well as those who perceive themselves as overweight are more likely to have health complaints compared to normal-weight peers^16,17^. However, little is known about health status and health complaints of underweight adolescents in high-income countries.

Children spend a considerable amount of time at school. Besides the main role of school in the academic development of children, it plays an important role in social development, physical and mental health. Understanding the factors associated with poor perception of school, absenteeism or low academic achievements is of great importance. For instance, it has been previously suggested that excessive weight may affect school attendance due to its negative effect on physical and mental health^18^. Thus, a recent meta-analysis has demonstrated that the odds of being absent from school was 27% and 54% higher among children with overweight and obesity respectively, compared to normal weight children^18^. In addition, several studies demonstrated that overweight school-aged children and adolescents are more exposed to bullying than their normal-weight counterparts^19,20^. In a US cross-sectional study of 4742 male and 5201 female public school students, associations of weight status with social relationships, school experiences, psychological well-being, and some future aspirations were observed^21^. In a recent study in adolescents (12–18 years old), better academic achievements were found among physically fit as well as normal weight participants compared to unfit and overweight/obese peers, respectively^22^.

Being overweight or underweight increases the risk of somatic and mental health problems, low self-esteem^1,23^, and may predispose to bullying, especially in children and adolescents. This, in turn, may lead to poor academic achievements, psychosomatic and school-related complaints. As such, the identification of school-related factors associated with body weight status may help to better understand which strategies can be developed to promote general well-being. To our knowledge, no large-scale study to date has systematically investigated the link between weight status, psychosomatic complaints and school-related characteristics. With this in mind, the present study involving 18,462 school-age students from Uppsala County, Sweden aimed to investigate the relationship between weight status and psychosomatic complaints (PSC) as well as school-related complaints with a focus on gender differences.

## Materials and methods

### Participants

A cohort of 29,106 adolescents aged 12–19 years attending grade 7, 9 and 2nd year of upper secondary school in the Swedish Uppsala County, were invited to participate anonymously and voluntarily in *the Life and Health Young Cross-sectional Survey*, conducted by the Uppsala County Council, Sweden in 2007, 2009, and 2011. The overall response rate was approximately 80 %. From the initial sample size (n = 29,106), 2314 students were excluded because of missing data on sex (n=119), weight, height (n=2145) or inappropriately high values for BMI (> 50 kg/m^2^, n=50); 7010 participants ware further excluded because they did not answer on one or several questions regarding the school-related characteristics. Further, 854 students were excluded due to missing information on the psychosomatic health complaints. We additionally excluded 466 individuals for the analysis because of missing covariates. In total, 18,462 participants (51% girls) had no missing values of measures of interest (including covariates) and were considered eligible for the present analysis.

Written information about the survey was sent to school principals. Adolescents and their legal guardians were informed about the purpose, content and administration of the survey as well as about its voluntary and anonymous nature. The survey was filled in during school hours in a test-like situation, i.e. with teachers present and no possibility to see others’ responses. All adolescents and their legal guardians had the possibility to decline participation without further explanation. As the students were surveyed anonymously and voluntarily and no biological material or sensitive personal information was collected, the written informed consent from a student or legal guardian, as well as ethical approval for the data analysis was not required (Ethical Review Act 2003:460, the act concerning the Ethical Review of Research Involving Humans, Sweden; Dnr 2012/244). Research was performed in accordance with relevant guidelines/regulations. The study followed the STROBE checklist.

### Assessment of weight status

Participant’s self-reported weight and height were used for BMI calculation (kg/m^2^). The international age- and gender specific BMI thresholds for children, developed by the International Obesity Task Force, were used to categorize subjects as “underweight” (corresponding to BMI of < 18.5 kg/m^2^ at age 18 years), “normal weight” (18.50–24.99 kg/m^2^ at age 18 years) and “overweight/obese” (≥ 25.0 kg/m^2^ at age 18 years)^24,25^. In the present analysis, overweight and obese adolescents were combined into the same category.

### Assessment of psychosomatic complaints (PSC)

The participants were asked how often they had health complaints during last three months. For this analysis, the following symptoms were used: headache; stomachache; pain in the back/hips; feeling nervous; difficulty to fall asleep, feeling low; pain in the neck/shoulders; and feeling anxious/worried. These types of complaints have previously been used as health indicators among school-age children and adolescents^26–28^. Response alternatives were on a 5-point Likert scale: “never”, “rarely”, “sometimes”, “often” and “always”. In the analyses with the separate PSCs, each item was dichotomized as never/ rarely/ sometimes *vs* often/ always. The scores were also summarized into a PSC index ranging from 0 to 32 points. The 90th percentile (P90) value of the entire sample was equal to 19 and used as a cut-off point to categorize students as having less complaints (below P90) and having many complaints (at/above P90). The internal reliability was high (Cronbach’s *α* = 0.83).

### Assessment of school-related complaints

In total, nine questions were used to assess school-related characteristics. Students were asked to choose one of the following alternatives (1) “corresponds exactly”; (2) “corresponds quite well”; (3) “corresponds to some extent”; (4) “corresponds quite bad”; and (5) “doesn’t correspond at all” to the first three statements if *school work is interesting*, if the student *has friends at school*, and if *parents encourage the student to do well at school*. Answers were dichotomized as yes (1–3) and no (4–5).

A fourth question was *how comfortable the student feels at school* with possible answers: (1) “Very comfortable”; (2) “pretty comfortable”; (3) “neither comfortable nor uncomfortable”; (4) “quite uncomfortable”; (5) “very uncomfortable”. Based on the answers, two categories were formed: feeling relatively good at school (1–3) and feeling bad at school (4–5). A fifth question, *academic failure* was measured by the question whether the student has failed any subject at school or not. Possible answers were as follows: (1) “no”, (2) “1–2 subjects”, (3) “3–4 subjects” or (4) “ ≥ 5 subjects”. Based on the answers, two categories were formed: failed no subject *vs.* failed at least one subject at school during the school year. Students were also asked if they *play truant* with possible answers on a 6-point scale: (1) “never”; (2) “yes, sometimes during the term”; (3) “yes, once a month”; (4) “yes, 2–3 times a month”; (5) “yes, once a week”; and (6) “yes, several times per week”. The responses were dichotomized as never/seldom (1–2) and play truant once a month or more often (3–6). The students were asked *how often they had been bullied by schoolmates during the term* and *how often they had bullied others*. Possible answers were: (1) “never”; (2) “yes, once”; and (3) “yes, few times”, which were dichotomized as not being bullied/not bullying another student(s) (1) and being bullied at school/bullying another student (s) at school (2–3). And finally, the participants answered the question regarding *how they see their future* with the answers on a 5-point scale as follows: (1) “I see my future as very bright”; (2) “I see my future as quite bright”; (3) “I see my future as neither bright nor dark”; (4) “I see my future as quite dark”; and (5) “I see my future as very dark”. The variable was classified as “expect to have a bright future” (1–3) and “don’t expect to have a bright future” (4–5).

### Assessment of covariates

Parents’ ethnic background was defined as Swedish (i.e. both parents are born in Sweden), mixed (i.e. only one parents is born in Sweden), or foreign (i.e. both parents are born abroad) based on question *“where were your parents born?”* Family household structure was categorized as living in a household with both parents *vs*. single-adult household/other household structure. Parents’ employment status was dichotomized as at least one parent was employed vs. unemployed/students/on sick leave/on disability pension/on parental leave/other. School location was defined either as situated in larger towns and municipalities near large towns or smaller towns/urban areas and rural municipalities. Year of survey was defined as 2007, 2009 or 2011.

### Statistical analysis

For the statistical analysis, SPSS version 24.0 (SPSS Inc, Chicago, IL) was used. Results of descriptive analyses are presented as means and standard deviations or numbers and percentages. A binary logistic regression analysis was utilized to examine the association between weight status (exposure variable) and the eight separate PSCs, and dichotomized overall PSC score (outcome variables) as well as weight status (exposure variable) and the nine school-related complaints (outcome variables). Potential confounders and intermediate variables were selected based on previously published results of other studies^8,27^ with the help of directed acyclic graphs^29^.

Multivariable analyses of the associations between weight status and PSCs was performed adjusted for adolescents’ age, parents’ ethnic background, family household structure, parents’ employment status, year of survey and school location. Two regression models were constructed to study the association between weight status and school-related complaints: Model A was adjusted for adolescents’ age, parents’ ethnic background, family household structure, parents’ employment status, year of survey and school location; model B was additionally adjusted for the potential intermediate variable, overall PSC index score. In this analysis, the results based on Model A were considered as the main findings. The data from boys and girls were analyzed separately and data from the underweight and overweight/obese adolescents were compared to those of normal-weight. The Benjamini–Hochberg method was applied to correct for multiple testing of all associations of weight status with PSC and school-related complaints. P values that passed a critical value corresponding to the False Discovery Rate (FDR) of 0.05 were considered as strong evidence of associations. For FDR correction, Stata (version 15; StataCorp, College Station, Texas) was used.

## Results

### Descriptive data

A total of 18,462 adolescents (49% boys and 51% girls) from Uppsala County, Sweden were included into the present analysis. Cohort characteristics, stratified by gender, are shown in Table 1. Being underweight was more prevalent among girls (11.6%) compared with boys (5%), while the proportion of overweight/obese was higher in boys (19.7%) than in girls (10.5%; *χ*2 = 502.9, *df* = 2, *P* < 0.001). The proportion of living with both parents was slightly higher in boys (66.5%) than in girls (64.6%, *χ*2 = 7.8, d*f* = 1, *P* = 0.005). A higher proportion of girls (63.8%) studied in larger cities and municipalities near large cities compared with boys (61%; *χ*2 = 16.2, d*f* = 1, *P* < 0.001). Additionally, girls had a higher frequency of PSC than boys (17.5% vs 3.8%, respectively, *χ*2 = 896.9, d*f* = 1, *P* < 0.0001) and a larger mean value of the psychosomatic complaints index (girls: 13.2, boys: 8.7, *P* < 0.001). No gender differences were found for age, grade, parents’ ethnic background and parents’ employment status. The proportion of PSCs among boys and girls is shown in Table 2. Tables 3 and 4 present the results of multivariable binary logistic regression analyses between weight status and PSCs, and weight status and school-related complaints, respectively.

**Table 1:** Socio-demographic characteristics of students included in the analyses.

	Boys	Girls
Total, n (%)	9078 (49.2)	9384 (50.8)
Age, years (SD)	15.91 (1.53)	15.90 (1.51)
**Weight status, n (%)**		
Underweight	454 (5.0)	1087 (11.6)
Normal-weight	6836 (75.3)	7311 (77.9)
Overweight/obese	1788 (19.7)	986 (10.5)
**Grade, n (%)**		
Grade 7	1340 (14.8)	1359 (14.5)
Grade 9	4077 (44.9)	4165 (44.4)
2 years upper secondary school	3661 (40.3)	3860 (41.1)
**Parent’s ethnic background, n (%)**		
Swedish	7071 (77.9)	7214 (76.9)
Mixed	1029 (11.3)	1107 (11.8)
Foreign	978 (10.8)	1063 (11.3)
**Household structure, n (%)**		
Living with both parents	6040 (66.5)	6060 (64.6)
Another family structure	3038 (33.5)	3324 (35.4)
**School location, n (%)**		
Larger towns and municipalities near large towns	5535 (61.0)	5991 (63.8)
Smaller towns/urban areas and rural municipalities	3543 (39.0)	3393 (36.2)
**Parent’s employment, n (%)**		
At least 1 parent is employed	8808 (97.0)	9069 (96.6)
Unemployed/students/on sick leave/ on disability pension/on parental leave/other	270 (3.0)	315 (3.4)
**Psychosomatic complaints, n (%)**		
Less complaints (below P90)	8730 (96.2)	7741 (82.5)
More complaints (at/above P90)	348 (3.8)	1643 (17.5)
Psychosomatic complaints index, Mean (SD)	8.71 (5.03)	13.17 (5.65)

*SD* standard deviation; *P90* 90th percentile.

**Table 2:** Proportion of self-reported psychosomatic complaints (PSC) experienced often or always among boys and girls.

	Boys N = 9078	Girls N = 9384	P-value ^a^
Headache, n (%)	808 (8.9)	2357 (25.1)	< 0.001
Stomach ache, n (%)	628 (6.9)	2130 (22.7)	< 0.001
Pain in the back/hips, n (%)	850 (9.4)	1742 (18.6)	< 0.001
Pain in the neck/shoulders, n (%)	970 (10.7)	2477 (26.4)	< 0.001
Feeling low, n (%)	714 (7.9)	1991 (21.2)	< 0.001
Feeling nervous, n (%)	796 (8.8)	2066 (22.0)	< 0.001
Difficulty to fall asleep, n (%)	1205 (13.3)	2129 (22.7)	< 0.001
Feeling anxious/worried, n (%)	662 (7.3)	2064 (22.0)	< 0.001

^a^Chi-square test.

**Table 3:** Associations of weight status and self-reported psychosomatic complaints (PSC).

	Boys (n = 9078)			Girls (n = 9384)		
	PSC rare, N (%)	PSC often, N (%)	OR (95%CI)	PSC rare, N (%)	PSC often, N (%)	OR (95%CI)
**Headache**						
Normal-weight	6273 (91.8)	563 (8.2)	1	5514 (75.4)	1797 (24.6)	1
Underweight	393 (86.6)	61 (13.4)	1.75 (1.32–2.33)*	810 (74.5)	277 (25.5)	1.06 (0.91–1.22)
Overweight/obese	1604 (89.7)	184 (10.3)	1.23 (1.03–1.47)*	703 (71.3)	283 (28.7)	1.17 (1.01–1.36)
**Stomach ache**						
Normal-weight	6397 (93.6)	439 (6.4)	1	5677 (77.7)	1634 (22.3)	1
Underweight	405 (89.2)	49 (10.8)	1.79 (1.31–2.45)*	815 (75.0)	272 (25.0)	1.16 (1.00–1.35)
Overweight/obese	1678 (92.2)	140 (7.8)	1.22 (1.00–1.49)	762 (77.3)	224 (22.7)	0.96 (0.82–1.13)
**Pain in the back/hips**						
Normal-weight	6237 (91.2)	599 (8.8)	1	5972 (81.7)	1339 (18.3)	1
Underweight	398 (87.7)	56 (12.3)	1.47 (1.10–1.97)*	896 (82.4)	191 (17.6)	0.96 (0.81–1.13)
Overweight/obese	1593 (89.1)	195 (10.9)	1.24 (1.05–1.47)*	774 (78.5)	212 (21.5)	1.14 (0.97–1.35)
**Pain in the neck/shoulders**						
Normal weight	6134 (89.7)	702 (10.3)	1	5412 (74.0)	1899 (26.0)	1
Underweight	386 (85.0)	68 (15.0)	1.54 (1.17–2.01)*	801 (73.7)	286 (26.3)	1.03 (0.89–1.19)
Overweight/obese	1588 (88.8)	200 (11.2)	1.07 (0.90–1.26)	694 (70.4)	292 (29.6)	1.14 (0.99–1.33)
**Feeling low**						
Normal weight	6343 (92.8)	493 (7.2)	1	5814 (73.5)	1497 (20.5)	1
Underweight	397 (87.4)	57 (12.6)	1.79 (1.33–2.41)*	858 (78.9)	229 (21.1)	1.04 (0.89–1.22)
Overweight/obese	1624 (90.8)	164 (9.2)	1.26 (1.05–1.52)*	721 (73.1)	265 (26.9)	1.37 (1.17–1.60)*
**Feeling nervous**						
Normal weight	6266 (91.7)	570 (8.3)	1	5747 (78.6)	1564 (21.4)	1
Underweight	392 (86.3)	62 (13.7)	1.74 (1.31–2.32)*	826 (76.0)	261 (24.0)	1.16 (1.00–1.35)
Overweight/obese	1624 (90.8)	164 (9.2)	1.05 (0.88–1.27)	745 (75.6)	241 (24.4)	1.15 (0.99–1.35)
**Difficulty to fall asleep**						
Normal weight	5978 (87.4)	858 (12.6)	1	5707 (78.1)	1604 (21.9)	1
Underweight	376 (82.8)	78 (17.2)	1.44 (1.12–1.86)*	822 (75.6)	265 (24.4)	1.15 (0.99–1.34)
Overweight/obese	1519 (85.0)	269 (15.0)	1.19 (1.02–1.38)	726 (73.6)	260 (26.4)	1.21 (1.04–1.42)*
**Feeling anxious/worried**						
Normal weight	6372 (93.2)	464 (6.8)	1	5751 (78.7)	1560 (21.3)	1
Underweight	400 (88.1)	54 (11.9)	1.84 (1.36–2.49)*	845 (77.7)	242 (22.3)	1.06 (0.91–1.24)
Overweight/obese	1644 (91.9)	144 (8.1)	1.14 (0.93–1.39)	724 (73.4)	262 (26.6)	1.26 (1.08–1.48)*

Logistic regression models adjusted for adolescent’s age, parents’ ethnic background, family household structure, parents’ employment status, school location, and year of survey.

*PSC rare* answer alternatives: never, seldom, sometimes; *PSC often* answer alternatives: often, always.

*CI* confidence interval; *PSC* psychosomatic complaints.

*Associations passed a critical P value corresponding to FDR of 0.05.

**Table 4:** Associations between weight status and school-related complaints among boys and girls.

	Boys (n = 9078)		Girls (n = 9384)	
	Model A	Model B	Model A	Model B
	OR (95%CI)	OR (95%CI)	OR (95%CI)	OR (95%CI)
**Academic failure**				
Underweight	1.44 (1.17–1.78)*	1.37 (1.11–1.69)	1.13 (0.97–1.33)	1.12 (0.96–1.32)
Overweight/obese	1.39 (1.24–1.56)*	1.38 (1.23–1.55)	1.71 (1.47–1.98)*	1.66 (1.42–1.93)
**School work is not interesting**				
Underweight	0.91 (0.72–1.13)	0.83 (0.66–1.05)	1.19(1.03–1.39)*	1.19 (1.02–1.38)
Overweight/obese	0.99 (0.88–1.12)	0.96 (0.85–1.09)	0.98 (0.83–1.15)	0.92 (0.78–1.09)
**Have no friends at school**				
Underweight	1.49 (0.91–2.44)	1.24 (0.75–2.06)	1.41 (0.96–2.07)	1.36 (0.92–2.00)
Overweight/obese	1.26 (0.94–1.69)	1.20 (0.89–1.62)	2.16 (1.54–3.03)*	1.96 (1.39–2.77)
**Parents don’t encourage to do well at school**				
Underweight	1.76 (1.09–2.84)*	1.55 (0.95–2.52)	1.67 (1.21–2.30)*	1.60 (1.16–2.23)
Overweight/obese	1.34 (1.00–1.80)	1.29 (0.96–1.74)	1.66 (1.20–2.29)*	1.52 (1.09–2.11)
**Does not feel good at school**				
Underweight	1.54 (1.05–2.26)	1.19 (0.80–1.78)	1.40 (1.09–1.81)*	1.37 (1.05–1.80)
Overweight/obese	1.49 (1.20–1.86)*	1.40 (1.12–1.75)	1.64 (1.28–2.12)*	1.47 (1.12–1.92)
**Play truant once a month or more often**				
Underweight	1.07 (0.84–1.36)	0.97 (0.76–1.25)	1.02 (0.87–1.20)	1.00 (0.85–1.18)
Overweight/obese	1.23 (1.08–1.40)*	1.20 (1.06–1.37)	1.00 (0.85–1.18)	0.95 (0.80–1.12)
**Being bullied at school**				
Underweight	1.30 (0.99–1.72)	1.12 (0.84–1.50)	1.02 (0.83–1.26)	1.01 (0.82–1.25)
Overweight/obese	1.29 (1.10–1.51)*	1.23 (1.04–1.44)	1.55 (1.28–1.89)*	1.45 (1.19–1.77)
**Bullying other student(s) at school**				
Underweight	0.93 (0.69–1.26)	0.84 (0.62–1.14)	1.24 (0.97–1.60)	1.23 (0.96–1.59)
Overweight/obese	1.29 (1.11–1.50)*	1.25 (1.07–1.46)	1.22 (0.94–1.59)	1.15 (0.88–1.50)
**Don’t expect to have a bright future**				
Underweight	1.46 (0.92–2.32)	1.03 (0.63–1.69)	1.24 (0.89–1.74)	1.15 (0.81–1.63)
Overweight/obese	1.13 (0.85–1.51)	1.03 (0.77–1.38)	1.59 (1.16–2.18)*	1.38 (0.99–1.92)

Normal weight is used as reference category.

Model A: Controlled for adolescent’s age, parents’ ethnic background, family household structure, parents’ employment status, school location, and year of survey.

Model B = Model A + psychosomatic complaints index.

*Associations passed a critical P value corresponding to FDR of 0.05 (Model A only).

### Psychosomatic complaints associated with weight status

Girls had higher prevalence of all eight PSC (headache, stomach ache, pain in the back/hips, pain in the neck/shoulders, feeling low, feeling nervous, difficulty to fall asleep and feeling anxious/worried) than boys (P < 0.0001, Table 2). The most common complaints among girls were pain in the neck/shoulders (26.4%) and headache (25.1%), whereas for boys it was difficulty to fall asleep (13.3%), pain in the neck/shoulders (10.7%) and pain in the back/hips (9.4%). After correction for multiple testing, multivariable analyses controlling for age, parents’ ethnic background, family household structure, parents’ employment status, year of survey and school location, revealed that underweight boys had a higher odds ratio for all eight PSC with odds ratios (OR) ranging from 1.44 to 1.84 than normal-weight boys (Table 3). Overweight/obese boys reported more often headache, pain in the back/hips, and feeling low compared with normal-weight boys. Underweight girls were more likely to report stomach ache than those of normal-weight. However, this association did not pass the multiple comparison threshold and no other associations between underweight among girls and PSCs were observed. Overweight/obese girls had higher odds feeling low, having difficulty to fall asleep and feeling anxious/worried compared with normal-weight girls (Table 3).

When analyzing the overall PSC index dichotomized as having fewer complaints (below P90) and having more complaints (at/above P90), our results revealed that underweight boys had higher odds to have more psychosomatic complaints in comparison with normal-weight boys (OR 2.52, 95% CI 1.75–3.63, P < 0.001, passed FDR correction). A weaker association was observed in underweight girls (OR 1.18, 95% CI 1.00–1.39, P = 0.049, did not pass FDR correction). Additionally, overweight/obese girls had higher odds to have more psychosomatic complaints in comparison with normal-weight girls: OR 1.23, 95% CI 1.04–1.45, P < 0.05, passed FDR correction (P for all BMI*sex interaction < 0.05).

### School-related complaints associated with weight status

Binary logistic regression analyses (Model A: adjusted for potential confounders; corrected for multiple testing) revealed that underweight boys were more likely to report that parents don’t encourage them to do well at school, and had higher odds of academic failure than normal weight boys (Table 4). The observed association remained significant in model B (additionally adjusted the overall PSC index score) only for academic failure. Compared with normal-weight, overweight/obese boys reported more often that they did not feel good at school, truancy, being bullied and bullying others at school, had higher odds of academic failure (Table 4). Underweight girls were more likely to report that school work is not interesting, to feel that parents don’t encourage them to do well at school, and not to feel good at school. The same pattern was observed when the model was additionally adjusted for PSC index (model B; Table 4). Compared with normal-weight girls, overweight/obese girls had higher odds of having no friends at school, to feel that parents don’t encourage them to do well at school, to not feel good at school, higher odds of academic failure, of being bullied at school, and not to expect to have a bright future (Table 4). The observed associations remained significant in the model B except for the variable regarding expectations about future (Table 4).

## Discussion

In the present study, being underweight or overweight/obese was adversely associated with psychosomatic complaints (PSC) and several school-related complaints including social interactions, academic failure, behavioral and emotional factors. Interestingly, different patterns were observed for boys and girls. In general, girls had a higher proportion of all psychosomatic complaints than boys. This is in line with several epidemiological studies which have demonstrated that adolescent girls tend to have higher frequency of psychosomatic symptoms than boys^26,30^. However, when taking weight status into account, underweight boys had higher odds to have all psychosomatic complaints than normal-weight counterparts, while no such associations were observed among underweight girls as compared with normal-weight girls. The link between being overweight/obese (vs normal-weight) and headache, pain in the back/hips and feeling low were observed among boys, whereas the association with complaints of feeling low, feeling anxious/worried and difficulty to fall asleep were found among girls.

Additionally, being underweight or overweight/obese was adversely associated with several school-related complaints. In general, there were more such associations for overweight/obese girls and boys compared with their normal-weight counterparts, than for the underweight adolescents compared with their normal-weight counterparts. Our findings based on a large adolescent cohort, provide additional evidence that weight status among adolescents may be one of the risk factors for psychosomatic and school-related complaints.

### Comparisons with other studies

Underweight is associated with malnutrition especially in low-income countries. However, in developed countries, underweight among children and adolescents is becoming a serious phenomenon that is sometimes underrated in contrast to considerable attention paid to overweight and obesity. Adolescents, especially girls, may experience external social pressure to be thin which in turn may lead to body dissatisfaction, extensive weight loss and underweight. Moreover, thinness is known to be associated with variety of adverse mental outcomes such as depression^8^, suicidal thoughts and attempts^9^, and poorer memory performance^31^. Results of the present study revealed important gender-specific associations of underweight with PSC with pronounced link of psychosomatic symptoms among underweight boys than normal-weight counterparts.

Studies of associations of weight status and school-related characteristics in adolescents are scarce. A cross-sectional study of 9943 US adolescents showed that underweight adolescents especially boys had poorer social relationships and school experiences than their average normal weight peers^21^. In agreement with these results, the current study demonstrated that underweight adolescents had higher odds to report less *parents’ encouragement to do well at school*. In addition, underweight boys were more likely to report *academic failure* whereas underweight girls had higher odds to report *not feeling good at school* and that *school work is not interesting* compared to normal weight counterparts.

In line with previous findings, our study revealed adverse associations between being overweight/obese and psychosomatic complaints as well as several school-related complaints. It was previously demonstrated that overweight or obesity in children and adolescents is often associated with development of psychological distress, low self-esteem, poor quality of life, discrimination and poorer academic performance^3,22,32–35^. For instance, a cross-sectional study of school-aged children aged 10 to 17 years showed that obese children had higher odds to have school problems and grade repetition^3^. In the same study, larger weight was also associated with higher rates of somatic and psychological disorders including attention deficit/hyperactivity disorder (ADHD), depression, learning problems, headaches^3^. Our results provide further support to the notion that overweight/obese (vs. normal-weight) adolescents are more likely to have *psychosomatic complaints*, such as *headache*, *feeling low* and *difficulties to fall asleep* than normal-weight counterparts, and emphasize a gender-specific nature of these associations. The present study also revealed that being overweight/obese among boys and girls was associated with *academic failure* and *not feeling good at school*, which is in line with previous reports^3,21,22,34^. These factors may adversely affect social interactions and future aspirations of overweight/obese adolescents. Thus, our findings demonstrated that overweight/obese girls were more likely to report that they *do not have friends at school*, *parents do not encourage them to do well at school* and *that they do not expect to have a bright future*.

Several studies reported that overweight school- aged children and adolescents are more exposed to bullying than their normal-weight counterparts^19,20,36^ which is consistent with results of the current study. Such a negative attitude towards overweight and obesity among adolescents may be in large extend explained by anti-fat stereotypes existing in modern societies. Additionally, results of the present study indicated that overweight/obese boys were more likely to *bully other students at school* and *play truant from school*.

The adverse association between being overweight/obese or underweight and several school-related complaints might be explained by psychological discomfort and poorer general well-being. Psychological problems such as anxiety, nervousness and mental stress can be associated with increased muscle tension^37,38^, which can contribute to pain, especially in the neck and shoulders^38^. Subjective psychosomatic complaints are not always related to a defined diagnosis or disease^39^ but may reflect psychological discomfort and impaired global well-being in childhood^40^. Possible mediation effect of PSC was observed in the current study. Thus, among boys the association between being underweight and feeling that *parents do not encourage to do well at school* was reduced to a non-significant level when adjusted to PSC. Similar pattern was observed among girls on relationship between being overweight/obese and *do not expect to have a bright future*.

Important strengths of our study are the large sample size and the possibility to adjust for a variety of confounders. Several limitations, however, apply to the present study. All measures in the present study were self-reported. For example, self-reported information on weight and height may lead to under- or over-estimation of body size. However, inappropriately high and low values for BMI^21^ were excluded from the analysis. Moreover, the proportion of underweight and overweight/obese was comparable to findings from previous studies among children and adolescents in European countries^41–43^. In addition, residual confounding by other factors not considered in the analysis of the present study (e.g., pubertal status, parents' education and BMI status) cannot be excluded. For example, information about menstrual cycle was not available and we do not known if reports of stomach ache are related to menstruation or not. Additionally, the cross-sectional nature of this study precludes any assumptions about cause and effect relationships. Thus, it is possible that poor educational and school-related psychosocial characteristics can lead to emotional stress and, as a consequence, to overeating or dietary restrictions among adolescents.

## Conclusions

In summary, our findings suggest that both underweight and overweight/obesity in adolescents are associated with higher odds of PSC as well as school-related complaints often in a gender-specific manner. The study highlights the importance of considering a detrimental impact of abnormal weight status on the psychological health, school experience and school achievements, which are important constituents of success and confidence in adult life.

## Data Availability

The datasets generated and/or analyzed in this current study are not publicly available based on the data-sharing agreement with Uppsala County.

## References

[1] Han JC, Lawlor DA, Kimm SY (2010). Childhood obesity. Lancet.

[2] Morrison KM, Shin S, Tarnopolsky M, Taylor VH (2014). Association of depression & health related quality of life with body composition in children and youth with obesity. J. Affect. Disord..

[3] Halfon N, Larson K, Slusser W (2013). Associations between obesity and comorbid mental health, developmental, and physical health conditions in a nationally representative sample of US children aged 10 to 17. Acad. Pediatr..

[4] Morris AM, Katzman DK (2003). The impact of the media on eating disorders in children and adolescents. Paediatr. Child Health.

[5] Rancourt D, McCullough MB (2015). Overlap in Eating Disorders and Obesity in Adolescence. Curr. Diab.Rep..

[6] Zhang Y, Wang R, Liu B, Sun L (2020). Weight in the mind: Weight perception and depressive symptoms in Chinese adolescents. J. Affect. Disord..

[7] Blashill AJ, Wilhelm S (2014). Body image distortions, weight, and depression in adolescent boys: Longitudinal trajectories into adulthood. Psychol. Men Masc..

[8] Revah-Levy A (2011). Association between Body Mass Index and depression: The "fat and jolly" hypothesis for adolescents girls. BMC Public Health.

[9] Whetstone LM, Morrissey SL, Cummings DM (2007). Children at risk: The association between perceived weight status and suicidal thoughts and attempts in middle school youth. J. Sch. Health.

[10] Patte KA, Livermore M, Qian W, Leatherdale ST (2021). Do weight perception and bullying victimization account for links between weight status and mental health among adolescents?. BMC Public Health.

[11] Haugland S, Wold B (2001). Subjective health complaints in adolescence–reliability and validity of survey methods. J. Adolesc..

[12] Garralda ME (1996). Somatisation in children. J. Child Psychol. Psychiatry.

[13] Kinnunen P, Laukkanen E, Kylma J (2010). Associations between psychosomatic symptoms in adolescence and mental health symptoms in early adulthood. Int. J. Nurs. Pract..

[14] Barkmann C (2015). Modelling trajectories of psychosomatic health complaints in children and adolescents: Results of the BELLA study. Eur. Child. Adolesc. Psychiatry.

[15] Hagquist C (2010). Discrepant trends in mental health complaints among younger and older adolescents in Sweden: An analysis of WHO data 1985–2005. J. Adolesc. Health.

[16] Castro-Pinero J (2012). Cardiorespiratory fitness and fatness are associated with health complaints and health risk behaviors in youth. J. Phys. Act. Health.

[17] Whitehead R (2016). Trends in adolescent overweight perception and its association with psychosomatic health 2002–2014: Evidence from 33 countries. J. Adolesc. Health.

[18] An R, Yan H, Shi X, Yang Y (2017). Childhood obesity and school absenteeism: A systematic review and meta-analysis. Obes. Rev..

[19] Brixval CS, Rayce SL, Rasmussen M, Holstein BE, Due P (2012). Overweight, body image and bullying: An epidemiological study of 11- to 15-years olds. Eur. J. Public Health.

[20] Danielsen YS (2012). Factors associated with low self-esteem in children with overweight. Obes. Facts.

[21] Falkner NH (2001). Social, educational, and psychological correlates of weight status in adolescents. Obes. Res..

[22] Martinez-Zamora MD, Valenzuela PL, Pinto-Escalona T, Martinez-de-Quel O (2020). The "Fat but Fit" paradox in the academic context: Relationship between physical fitness and weight status with adolescents' academic achievement. Int. J. Obes. (Lond).

[23] Drosopoulou G (2021). Psychosocial health of adolescents in relation to underweight, overweight/obese status: The EU NET ADB survey. Eur. J. Public Health.

[24] Cole TJ, Bellizzi MC, Flegal KM, Dietz WH (2000). Establishing a standard definition for child overweight and obesity worldwide: International survey. BMJ.

[25] Cole TJ, Flegal KM, Nicholls D, Jackson AA (2007). Body mass index cut offs to define thinness in children and adolescents: International survey. BMJ.

[26] Aslund C, Starrin B, Nilsson KW (2010). Social capital in relation to depression, musculoskeletal pain, and psychosomatic symptoms: A cross-sectional study of a large population-based cohort of Swedish adolescents. BMC Public Health.

[27] Carlerby H, Viitasara E, Knutsson A, Gillander Gadin K (2011). Subjective health complaints among boys and girls in the Swedish HBSC study: Focussing on parental foreign background. Int. J. Public Health.

[28] Conden E, Leppert J, Ekselius L, Aslund C (2013). Type D personality is a risk factor for psychosomatic symptoms and musculoskeletal pain among adolescents: A cross-sectional study of a large population-based cohort of Swedish adolescents. BMC Pediatr..

[29] Textor J, Hardt J, Knuppel S (2011). DAGitty: A graphical tool for analyzing causal diagrams. Epidemiology.

[30] Ostberg V, Alfven G, Hjern A (2006). Living conditions and psychosomatic complaints in Swedish schoolchildren. Acta Paediatr..

[31] Gunstad J (2008). Body mass index and neuropsychological function in healthy children and adolescents. Appetite.

[32] Forste R, Moore E (2012). Adolescent obesity and life satisfaction: Perceptions of self, peers, family, and school. Econ. Hum. Biol..

[33] Carey FR, Singh GK, Brown HS, Wilkinson AV (2015). Educational outcomes associated with childhood obesity in the United States: Cross-sectional results from the 2011–2012 National Survey of Children's Health. Int. J. Behav. Nutr. Phys. Act.

[34] Ma L (2020). Overweight and Obesity impair academic performance in adolescence: A national cohort study of 10,279 adolescents in China. Obesity (Silver Spring).

[35] Livermore M, Duncan MJ, Leatherdale ST, Patte KA (2020). Are weight status and weight perception associated with academic performance among youth?. J. Eat Disord..

[36] Lian Q (2018). The association between chronic bullying victimization with weight status and body self-image: A cross-national study in 39 countries. PeerJ.

[37] Van Galen GP, Muller ML, Meulenbroek RG, Van Gemmert AW (2002). Forearm EMG response activity during motor performance in individuals prone to increased stress reactivity. Am. J. Ind. Med..

[38] Lundberg U (2003). Psychological stress and musculoskeletal disorders: Psychobiological mechanisms. Lack of rest and recovery greater problem than workload. Lakartidningen.

[39] Garralda ME (1992). A selective review of child psychiatric syndromes with a somatic presentation. Br. J. Psychiatry.

[40] Petanidou D (2012). Identifying the sociodemographic determinants of subjective health complaints in a cross-sectional study of Greek adolescents. Ann. Gen. Psychiatry.

[41] Vaezghasemi M, Lindkvist M, Ivarsson A, Eurenius E (2012). Overweight and lifestyle among 13–15 year olds: A cross-sectional study in northern Sweden. Scand. J. Public Health.

[42] Gurzkowska B (2014). The relationship between selected socioeconomic factors and basic anthropometric parameters of school-aged children and adolescents in Poland. Eur. J. Pediatr..

[43] Sivertsen B, Pallesen S, Sand L, Hysing M (2014). Sleep and body mass index in adolescence: Results from a large population-based study of Norwegian adolescents aged 16 to 19 years. BMC Pediatr..

